# Daily Step Count Is Associated with Frailty Status in Veterans Presenting for Major Operations

**DOI:** 10.3390/geriatrics11040088

**Published:** 2026-07-16

**Authors:** Jessica Y. Rove, Yi Su, Jennifer E. Stevens-Lapsley, Hillary D. Lum, Thomas N. Robinson

**Affiliations:** 1 Department of Surgery, University of Colorado Anschutz, Aurora, CO 80045, USA; 2 Adult and Child Center for Outcomes Research and Delivery Science, University of Colorado Anschutz, Aurora, CO 80045, USA; 3 Department of Physical Medicine and Rehabilitation, University of Colorado Anschutz, Aurora, CO 80045, USA; 4 Department of Veterans Affairs Eastern Colorado Geriatric Research Education and Clinical Center, Aurora, CO 80045, USA; 5 Department of Medicine, University of Colorado Anschutz, Aurora, CO 80045, USA; hillary.lum@cuanschutz.edu

**Keywords:** daily step count, geriatric surgery, postoperative recovery

## Abstract

**Background/Objectives**: Frailty is an under-recognized preoperative risk factor for poor surgical outcomes. Recovery of preoperative ambulation levels one month after surgery represents an important patient-centered outcome. The purpose of this study was to (1) identify a preoperative daily step count threshold associated with frailty, and (2) characterize the one-month ambulation recovery (steps/day) of older adults (≥60 years) with and without frailty following major elective inpatient operations. **Methods**: This single-center observational pilot study included older adults undergoing elective inpatient operations at a Veterans Affairs Hospital. Frailty was assessed before surgery. Ambulation (steps/day) was measured continuously from ≥3 full days before surgery, to at least 28 days after surgery, using a wrist-worn accelerometer. Sensitivity analysis identified a step count threshold associated with frailty. The primary outcome was the percentage of daily steps recovered at 28 days. **Results**: The average age of participants (*n* = 109) was 69 years, and 37.6% (41/109) were identified as prefrail or frail. Preoperative steps/day were lower in the frail cohort (3322.00 [2654.70, 5483.20] versus 4993.95 [3540.90, 6599.97], *p* < 0.05). Sensitivity analysis showed 4137 steps/day to be associated with preoperative frailty, and this remained significant in multivariate analysis. At 28 days, the percentage of daily steps recovered did not differ between groups (72.57% [49.15, 92.06] overall). **Conclusions**: Preoperative frailty is associated with lower daily step counts (<4137 steps/day), identifying individuals at increased likelihood of frailty in this older Veteran cohort undergoing major surgery. The median daily step count, regardless of frailty status, was <5000 steps/day, consistent with a sedentary lifestyle, and likely too low to show a difference in one-month ambulation recovery. This finding underscores the need to explore walking interventions in older Veterans awaiting surgery.

## 1. Introduction

Older adults (≥60 years) undergo nearly half of the 3.7 million operations performed annually in the United States [1]. Frailty is common in older adults prior to both cardiac and non-cardiac operations [2,3]. Frailty is a geriatric syndrome defined as decreased physiologic reserve and resistance to stressors, distinct from comorbidity and disability [4]. Frailty assessment generally includes a measurement of physical activity, strength, cognition, and nutrition [5,6]. Prefrail and frail status are closely associated with poor postsurgical outcomes. Multiple studies find a close relationship to frailty and increased serious postoperative complications, prolonged hospital stays, disability, higher healthcare costs, and death [2,7,8,9,10]. Preoperative assessment of frailty is recommended in guidelines from the American Geriatric Society and American College of Surgeons [4].

Low activity levels are a vital health marker and are closely related to poor health, disability, and the occurrence of geriatric syndromes [11,12]. Health-related quality of life is better in older adults who walk more steps/day [13]. Low daily step counts are associated with mortality, and higher daily step counts progressively decreased the risk of all-cause mortality and dementia [14,15].

Improving postoperative outcomes in patients with frailty is a priority, but efficacy data for preoperative, real-world interventions remain a crucial evidence gap [16,17]. Walking before surgery may be a measurable and modifiable risk factor in frail patients [18,19]. The purpose of this study was to (1) identify a baseline daily step count threshold associated with preoperative frailty, and (2) define the one-month ambulation recovery (steps/day) of older adults (≥60 years) with and without frailty following major elective inpatient operations. We hypothesized that preoperative frailty would be associated with delayed daily step count recovery in older adults compared to non-frail individuals. 

## 2. Materials and Methods

### 2.1. Study Design

This single-center, prospective, observational pilot study included patients 60 years and older scheduled for elective inpatient operations (abdominal and cardiothoracic) between November 2016 and July 2019 at a referral Veterans Affairs Medical Center. Recruitment employed convenience sampling. Patients were screened in preoperative clinics by study staff. Exclusion criteria were patients who were non-ambulatory (e.g., wheelchair-bound) and elective operations scheduled without at least 3 full calendar days to establish community preoperative baseline ambulation levels prior to their operation. Written informed consent was obtained from patients prior to participation (Colorado Multi-Institutional Review Board #16-1776, last updated 27 April 2020). The planned recruitment of over 100 patients in this pilot study aims to demonstrate the feasibility of completing geriatric preoperative assessment, tracking daily step count before and after surgery in an older Veteran cohort, and evaluating clinically relevant outcomes to guide power analyses for future work.

### 2.2. Measuring Step Counts

Preoperative and postoperative ambulation (recorded as steps per calendar day) was measured by a wrist-worn accelerometer activity tracker (Vivofit 3, Garmin Ltd., Olathe, KS, USA). This device is validated for tracking steps with <1% deviation from a video observation of steps taken, which is the current gold standard [20]. Before and after surgery, patients were instructed to continue their usual activities. Target daily step counts were not discussed. The activity trackers were water-resistant and did not require charging during the study period. Patients were instructed to wear the watch continuously as able. All activity trackers were synced to a smartphone application that allowed data to be downloaded during clinically indicated encounters: at the time of surgery, discharge, and follow-up. Postoperative ambulation was recorded for at least 28 days. Inpatient and home recovery days were not treated differently; the postoperative period was treated as a continuum. 

Average preoperative daily step count was calculated by taking the total steps observed in the days between study enrollment and the day of surgery (minimum 3 days and up to 10 days), divided by the number of days. Daily step count measured on the day of enrollment and the day of surgery was excluded from analysis because patients did not have a full day to track steps. 

### 2.3. Study Variables and Analysis

The predictor variable was prefrail or frail versus non-frail. Seven baseline frailty traits were assessed: Katz score less than or equal to 5, Timed Up-and-Go test greater than or equal to 15 s, Charlson Index greater than or equal to 3, anemia less than 35%, Mini-Cog score less than or equal to 3, albumin less than 3.4 g/dL, and 1 or more falls within 6 months. Patients were categorized by the number of positive traits as follows: non-frail: 0 to 1 trait, prefrail: 2 to 3 traits, and frail: 4 or more traits [6]. 

Preoperative, intraoperative, and postoperative clinical variables were prospectively recorded. Preoperative comorbidities and postoperative complications were defined using the standardized National Surgical Quality Improvement Program definitions (https://www.facs.org/quality-programs/data-and-registries/acs-nsqip/participant-use-data-file/, accessed 1 May 2026). Preoperative variables recorded included baseline demographics, comorbidities, the Charlson Comorbidity Index [21], an American Society of Anesthesiology (ASA) score [22], and depression score using Personal Health Questionnaire-2 (PHQ-2). Intraoperative variables included length of operation, procedure type, and blood loss. Postoperative variables included hospital length of stay, surgical complications (composite of one or more cardiac, pulmonary, renal, infectious, and thrombotic complications), and readmissions. 

Baseline characteristics were summarized as medians with interquartile ranges for continuous variables and counts with percentages for categorical variables. Variables were compared between frailty groups using Wilcoxon rank sum tests and Fisher’s exact tests, respectively, reported in Table 1. The optimal cutoff for preoperative step counts associated with prefrail/frail status was identified using logistic regression with frailty as the outcome and maximizing Youden’s J statistic. Univariate logistic regression analyses were used to examine associations between explanatory variables and frailty; variables with a *p*-value < 0.05 and considered clinically relevant were included in a multivariable logistic regression model. Adjusted odds ratios with 95% CIs were reported in Table 2. All tests were 2-sided, and *p*-values < 0.05 were considered statistically significant. Missing data were not imputed and were analyzed as observed. All calendar days with steps recorded were included. Patient days with missing step count data were excluded from statistical analysis. Device usage rate was defined as days with steps recorded divided by all days with the step tracker and reported separately for the preoperative and postoperative periods. All analyses were conducted using R.4.3.2 [23].

### 2.4. Outcomes

The primary outcome of this pilot study was the percentage of daily steps recovered at 28 days. Postoperative walking recovery (steps/day) was represented both as an absolute value and as a percentage of preoperative baseline ambulation for each postoperative day using the patient as their own reference. To assess where a patient was in comparison to their own baseline, the number of steps per day for each postoperative day was divided by the patient’s preoperative average number of steps. For example, if a participant averaged 10,000 steps daily preoperatively, the finding of 4000 steps on postoperative day #7 is reported as a recovery of 40% of their baseline.

## 3. Results

A total of 109 adults ≥ 60 years or older were consecutively enrolled in this pilot study. The average age was 69 years, and 95.4% were male. The patients were stratified by frailty status, with 68 patients meeting criteria for non-frail and 41 patients meeting criteria for prefrail/frail. 

### 3.1. Baseline Characteristics

Preoperative characteristics are summarized in Table 1. The prefrail/frail patients had more cognitive impairment, more diabetes, a higher Charlson comorbidity index, lower Timed Up-and-Go, worse independent function, more falls, and more depression. The average preoperative steps per day were significantly different between non-frail and prefrail/frail patients, 4993.95 [3540.90, 6599.97] and 3322.00 [2654.70, 5483.20] (*p* < 0.05). 

### 3.2. Postoperative Outcomes

Postoperative outcomes are summarized in Table 1. Prefrail/frail patients were more likely to undergo abdominal surgery as opposed to cardiothoracic surgery and had shorter operative times. There were no significant differences between prefrail/frail and non-frail patients in length of stay, delirium, discharge to facility, major postoperative complications, hospital readmission, median steps per postoperative week, or median percent ambulatory recovery by postoperative week. 

### 3.3. Daily Step Count Cutoff Associated with Frailty

Device usage was 98% and 94% in the preoperative and postoperative periods, respectively. Preoperative daily step count was assessed over a mean and median of 8.3 and 10 days, respectively (range 3–10 days). Sensitivity analysis of preoperative daily step counts found that 4137 steps/day was the cutoff associated with prefrail/frail status, yielding a sensitivity of 63% and specificity of 69%. Patients below this threshold had higher odds of frailty (OR 2.81, 95% CI 1.08–7.73; *p* = 0.01).

Univariate logistic regression analyses were used to examine the association between frailty and preoperative daily steps < 4137 steps/day, among other variables. Variables with *p*-value < 0.05 and not included in the baseline frailty assessment were included in a multivariable logistic regression model. Preoperative daily step count < 4137 steps/day, white race, higher depression score, and diabetes were unique variables associated with frailty (Table 2).

## 4. Discussion

In a cohort of older Veterans undergoing major cardiac and non-cardiac surgery, prefrail/frail status was associated with walking less than 4137 steps/day, representing a threshold associated with increased likelihood of frailty. This value falls within a range consistent with sedentary activity levels. These are the first contemporary pilot data to associate daily step count and frailty in an older adult Veteran population requiring major surgery. These results corroborate a large, community-based study showing that frailty in older adults is associated with 4000 steps [18]. One-month ambulation recovery was low in both cohorts, with medians of 71.65% and 76.34% in the prefrail/frail versus non-frail patients, respectively. In patients who walked <4137 steps/day, the average daily step count one month after surgery was critically low, averaging 1612 steps. Future studies will require following patients longitudinally for longer than 28 days to observe if, over time, there is a difference in recovery trajectories between prefrail/frail and non-frail patients.

The median daily step count observed, regardless of frailty status, was <5000 steps/day, consistent with a sedentary lifestyle [24]. This low baseline activity likely limited our ability to detect differences in one-month recovery between groups. When baseline activity is this low, relative recovery metrics may be less sensitive to differences in physiologic reserve. Health-related quality of life is better in older adults who walk more steps/day [13]. Low daily step counts are associated with mortality, and higher daily step counts have been associated with decreased risk of all-cause mortality and dementia [14,15]. Importantly, the data from our study highlight an opportunity to target walking interventions in older Veterans. Geriatrics walking interventions in Veterans have been shown to increase daily step counts and improve health outcomes [25,26]. These programs may serve as a model for prehabilitation in Veterans awaiting surgery. Future studies will need to determine if a preoperative walking intervention has a meaningful impact on postoperative outcomes, including physical function, cognition, and mental health. 

Neither frailty nor walking less than 4137 steps/day was associated with any postoperative outcomes typically associated with frailty. Delirium, postoperative complications, length of stay, and hospital readmission were similar in both groups. This study enrolled older Veterans undergoing major surgery, both abdominal and thoracic, cardiac and non-cardiac, minimally invasive and non-minimally invasive. Though operative approach and operative type were balanced between groups, postoperative differences associated with frailty may still be masked by differences in postoperative course related to surgery type and approach. For example, cardiac surgery patients leave the operating room intubated and recover in the intensive care unit, while a patient undergoing a laparoscopic colon resection is typically extubated and does not require critical care during recovery. In addition, postoperative data after one month were not collected, which limits examining longer-term recovery trajectories associated with frailty or baseline step count. 

Prior work has demonstrated that frailty is a risk factor for delirium and postoperative decline in cognitive function [9,10]. Delirium screening at our institution is with the Confusion Assessment Method for the Intensive Care Unit (CAM-ICU) [27]. CAM-ICU has been validated with a sensitivity of >93% and specificity of >98%, but studies have demonstrated that routine clinical CAM-ICU assessment misses a substantial proportion of delirium compared to assessment by trained research staff [28]. Continued training of bedside nurses may be necessary to improve delirium detection at our institution [29].

## 5. Limitations

This single-center observational study has multiple limitations. As a pilot exploration, this study was not powered to determine causality, and the sample size limits our ability to assess a dose–response of daily step count related to frailty status or postoperative outcomes. Future work will focus on comparing comparable operations to better characterize daily step count and physical activity by age, functional status, and pathology. Though a single-center design limits the generalizability of results, anesthesia and other care practices followed institutional protocols, which minimizes some variability. Patients in this study reflect a Veteran hospital demographic—predominantly white, non-Hispanic, and male—and may not be representative of a more diverse population. Sex-stratified analyses were not performed due to the small numbers of women. Despite these limitations, this pilot study is critical to informing power calculations for future work on frailty-related risk stratification and walking interventions in Veterans undergoing major surgery.

## 6. Conclusions

In this single-center pilot study, preoperative frailty was associated with walking <4137 steps/day in this older Veteran cohort undergoing major surgery. Though daily step counts in this Veteran population were consistent with a sedentary lifestyle, and thus, likely too low to show a difference in one-month postoperative ambulation recovery, this finding highlights an opportunity for future work to explore walking interventions in older Veterans awaiting surgery.

## Figures and Tables

**Table 1:** Preoperative characteristics, operative variables, and postoperative outcomes.

Variable	Non-Frail *n* = 68	Prefrail/Frail *n* = 41	Overall *n* = 109	*p*-Value
**Age (years)**	69.00 [64.75, 72.00]	71.00 [67.00, 73.00]	69.00 [65.00, 72.00]	0.1557
**Male Gender**	64 (94.1%)	40 (97.6%)	104 (95.4%)	0.6482
* Mini-Cog Score	5.00 [4.00, 5.00]	3.00 [2.00, 4.00]	4.00 [3.00, 5.00]	<0.01
**Cognitively impaired** (Mini-Cog < 3)	5 (7.4%)	14 (34.1%)	19 (17.4%)	<0.01
**Race**				
Black	3 (4.4%)	4 (9.8%)	7 (6.4%)	0.0688
American Indian/Alaska Native	1 (1.5%)	1 (2.4%)	2 (1.8%)	
Hispanic or Latino ethnicity	4 (5.9%)	6 (14.6%)	10 (9.2%)	
White	58 (85.3%)	28 (68.3%)	86 (78.9%)	
Asian	0 (0%)	2 (4.9%)	2 (1.8%)	
Missing	2 (2.9%)	0 (0%)	2 (1.8%)	
**Body mass index**	27.00 [23.00, 30.25]	29.00 [25.00, 32.00]	28.00 [24.00, 31.00]	0.2324
**Diabetes**	14 (20.6%)	20 (48.8%)	34 (31.2%)	<0.01
**Chronic obstructive pulmonary disease**	8 (11.8%)	5 (12.2%)	13 (11.9%)	1
**Creatinine, mg/dL**	1.10 [0.90, 1.20]	1.10 [0.90, 1.35]	1.10 [0.90, 1.30]	0.3996
* Albumin	3.80 [3.70, 4.00]	3.50 [3.20, 3.80]	3.70 [3.50, 3.90]	<0.01
Missing	3 (4.4%)	0 (0%)	3 (2.8%)	
* Charlson Comorbidity Index > 3				
Yes	7 (10.3%)	16 (39.0%)	23 (21.1%)	<0.01
No	60 (88.2%)	25 (61.0%)	85 (78.0%)	
Missing	1 (1.5%)	0 (0%)	1 (0.9%)	
**ASA score ≥ 3**	55 (80.9%)	36 (87.8%)	91 (83.5%)	0.4305
**Depression Score (PHQ-2**)	0.00 [0.00, 0.00]	1.00 [0.00, 2.00]	0.00 [0.00, 1.00]	<0.01
* Timed Up-and-Go (seconds)	9.00 [9.00, 11.00]	12.00 [9.00, 14.00]	10.00 [9.00, 12.00]	<0.01
Missing	7 (10.3%)	1 (2.4%)	8 (7.3%)	
* Katz ADL = 6	68 (100%)	35 (85.4%)	103 (94.5%)	<0.01
* Fall in past 6 months	3 (4.4%)	16 (39.0%)	19 (17.4%)	<0.01
**Average preoperative steps/day**	4993.95 [3540.90, 6599.97]	3322.00 [2654.70, 5483.20]	4448.70 [2981.60, 6303.90]	<0.05
**Operation Type**				
Abdominal	47 (69.1%)	36 (87.8%)	83 (76.1%)	<0.05
Cardiothoracic	21 (30.9%)	5 (12.2%)	26 (23.9%)	
**Operative Time (min)**	226.50 [164.50, 346.75]	174.00 [123.00, 247.00]	210.00 [151.00, 325.00]	<0.01
**Operative Approach**				
Laparoscopic	20 (29.4%)	8 (19.5%)	28 (25.7%)	0.3902
Open	37 (54.4%)	28 (68.3%)	65 (59.6%)	
Robotic	11 (16.2%)	5 (12.2%)	16 (14.7%)	
**Operative blood loss (mL)**	87.50 [31.25, 237.50]	50.00 [40.00, 200.00]	67.50 [35.00, 212.50]	0.4928
Missing	10 (14.7%)	3 (7.3%)	13 (11.9%)	
**Major postoperative complication**	7 (10.3%)	3 (7.3%)	10 (9.2%)	0.74
**Postoperative delirium**	9 (13.2%)	8 (19.5%)	17 (15.6%)	0.4213
**Length of stay (days)**	6.00 [3.00, 9.00]	6.00 [4.00, 8.00]	6.00 [3.00, 9.00]	0.8924
**Discharge to facility**				
Yes	2 (2.9%)	3 (7.3%)	5 (4.6%)	0.3657
No	65 (95.6%)	38 (92.7%)	103 (94.5%)	
Missing	1 (1.5%)	0 (0%)	1 (0.9%)	
**Hospital readmission**	8 (11.8%)	6 (14.6%)	14 (12.8%)	0.7697
**POD 7 Steps/Day (Median [IQR])**	1162.50 [437.75, 2495.00]	1184.00 [445.00, 2149.00]	1177.00 [439.00, 2355.00]	0.6843
**POD 7% Recovery (Median [IQR])**	24.99 [10.17, 52.42]	29.25 [16.53, 47.67]	27.03 [14.79, 50.59]	0.8
**POD 14 Steps/Day (Median [IQR])**	2003.00 [949.00, 3404.00]	1165.00 [375.00, 3848.00]	1860.00 [596.00, 3410.00]	0.0993
**POD 14% Recovery (Median [IQR])**	43.12 [26.49, 60.89]	31.21 [18.04, 67.56]	42.14 [20.14, 64.86]	0.3661
**POD 21 Steps/Day (Median [IQR])**	2581.00 [1380.00, 4280.75]	1703.00 [571.75, 3195.75]	2367.50 [1082.25, 3997.00]	<0.05
Missing	4 (5.9%)	3 (7.3%)	7 (6.4%)	
**POD 21% Recovery (Median [IQR])**	59.27 [32.97, 75.08]	45.58 [33.11, 70.77]	57.29 [32.93, 73.40]	0.4403
Missing	4 (5.9%)	3 (7.3%)	7 (6.4%)	
**POD 28 Steps/Day (Median [IQR])**	2786.00 [1455.50, 4973.50]	2451.00 [1007.50, 4135.00]	2543.00 [1144.00, 4852.50]	0.4766
Missing	9 (13.2%)	6 (14.6%)	15 (13.8%)	
**POD 28% Recovery (Median [IQR])**	76.34 [37.46, 97.91]	71.65 [58.19, 88.77]	72.57 [49.15, 92.09]	0.888
Missing	9 (13.2%)	6 (14.6%)	15 (13.8%)	

(*) Indicates this characteristic is included in assessment of frailty [6].

**Table 2:** Univariate and multivariate analysis of variables associated with frailty.

Univariate, Variable	OR (95% CI)	*p*-Value	Multivariate, Variable	OR (95% CI)	*p*-Value
* Mini-Cog	0.4648 (0.315–0.6544)	0	Age	1.0779 (0.9854–1.1871)	0.1016
* Timed Up-and-Go	1.4709 (1.2084–1.8487)	0	White race	0.1939 (0.0525–0.6308)	0.0058
Depression score	2.6466 (1.5483–4.7449)	0.0003	Depression score	2.2631 (1.2134–4.4405)	0.0098
* Charlson Comorbidity Index > 3	5.2196 (2.0199–14.6207)	0.0006			
Preoperative step count < 4137 steps/day	3.7772 (1.7075–8.6429)	0.0009	Preoperative step count < 4137 steps/day	2.8144 (1.077–7.73)	0.0347
* Albumin	0.1561 (0.0427–0.4897)	0.0011			
Diabetes	3.5838 (1.5679–8.4374)	0.0024	Diabetes	3.5481 (1.3044–10.1913)	0.013
Operation type: cardiothoracic	0.3329 (0.1085–0.8827)	0.0262	Operation type: cardiothoracic	0.546 (0.1439–1.7766)	0.3233

(*) Indicates this characteristic is included in assessment of frailty [6].

## Data Availability

Individual patient-level data is unavailable due to privacy restrictions as stated in the informed consent.

## Abbreviations

The following abbreviations are used in this manuscript:

ADL	Activities of daily living
ASA	American Society of Anesthesiology
POD	Postoperative day
PHQ-2	Personal Health Questionnaire-2
